# Suitability of agronomic water saving in karst areas and its enlightenment in the karst desertification control

**DOI:** 10.1016/j.heliyon.2024.e32568

**Published:** 2024-06-06

**Authors:** Qinglin Wu, Lan Wang

**Affiliations:** a School of Karst Science, Guizhou Normal University / State Engineering Technology Institute for Karst Desertification Control, Guiyang, 550001, China; b School of Foreign Languages, Guizhou Normal University, Guiyang, 550001, China

**Keywords:** Ecological industry, Karst drought, Suitability, Water use efficiency

## Abstract

The special "dual" hydrogeological structure in karst areas causes rainfall easily "leaking" into the ground, resulting a unique "karst drought". In these areas, drought and insufficient water resources seriously restrict the sustainable development of agriculture. In order to restore the ecology of karst desertification, develop ecological industries, improve the utilization efficiency of water resources, and advance water-saving agriculture in such areas, literature review method was applied to discuss the suitability of agronomic water-saving measures in karst areas. The results are as follows. (1) Agronomic water-saving measures including tillage, mulching, water-fertilizer coupling, chemical regulation, crop allocation and deficit irrigation can all enhance the crop WUE. For example, deep tillage and deep loosening increased the WUE by 15.1 % and 15.9 % respectively. The WUE of spring wheat under straw mulching increased by 17.17 %–43.01 % compared with that under mulching film. Increased density of intercropping corn and wheat saved 9.85 % of water. (2) The cultural or natural particularity of karst areas limits the application of all agronomic water-saving measures in karst areas, and therefore choices and adjustments are necessary according to local conditions: ① No tillage should be adopted because of the high output of labor force; ② straw mulching need to be crushed; ③ the coupling of water and fertilizer reaches better effect when applied to crops several hours before rainfall; ④ the shallow soil layer and the complexity of preparing water retaining agent make it unsuitable to use water retaining agent; ⑤ agroforestry with dwarf and dense planting is more suitable; ⑥ crop deficit irrigation can be carried out by using ecological small pools. Based on the above results, proposes are offered in the following. First, it is necessary to construct the optimal model of regional water and fertilizer coupling in karst areas, and apply composite agronomic water-saving measures. Second, it is suggested to establish a model of coordinating forest, grain and grass, and vigorously develop ecologically derivative agroforestry. Third, there is a necessity to strengthen the research and development of technology about soil and water leakage monitoring and resistance, and intensify studies on "five waters" transformation at the basin scale. The research results and implication are an important reference for developing water-saving agriculture, solving the shortage of agricultural water resources, ensuring the sustainability of agriculture and improving farmers' living standards. Rational use of agronomic water-saving measures is of great significance to enhance the utilization efficiency of water resources and boost regional economy in karst desertification areas.

## 1 Introduction

The global karst area is about 22 million km^2^, accounting for 15 % of the land area [1]. Carbonate rocks crop out over approximately 10 % of the world's land area, and roughly 20–25 % of the global population depends largely or entirely on groundwater in karst regions [2]. In China, karst areas are mainly distributed in its southwest regions, centered on the Guizhou plateau. It is the widest area with exposed carbonate rocks in the world (more than 0.54 million km^2^), being home to 220 million people [3,4]. It is also one of the most ecologically fragile regions in the world [5]. Karst desertification is the primary cause of vulnerable ecosystem here. It is an extreme manifestation of land degradation and the result of ecological degradation formed under unique hydrological and geological conditions [6]. Land surface presents high permeability in the karst area, which determines the poor capacity of water collection by the surface and that water resources are easy to leak into the ground through cracks [7], forming the distribution characteristics of water resources with poor surface water, and presenting a unique phenomenon of ‘karst drought’. Under ‘karst drought’, crops on the surface show drought characteristics within a week after rainfall, urgently requiring the application of agronomic water-saving measures in karst areas. Agronomic water-saving measures improves the utilization rate of water resources by inhibiting soil water evaporation and crop luxury transpiration [8,9]. It is characterized by less investment, operation facility and easy acceptance by farmers, serving as a key technology for comprehensively controlling soil and water loss in karst desertification and having great significance to the development of water-saving agriculture [10,11]. The survey data of farmers shows 91.9 % of farmers willing to adopt agronomic water-saving technology [12].

Agronomic measures are divided into tillage [13], mulching [14], water-fertilizer coupling [15], chemical regulation [16], and crop allocation (such as intercropping [17] and agroforestry [18]), deficit irrigaion [19]. Their purpose is to obtain more crop yield, increase soil fertility, and improve soil ecological environment and crop quality [20]. At present, a large body of studies have explored the various benefits of agronomic measures, such as crop yield, soil improvement, environmental benefits, water and fertilizer use efficiency, and reduction of heavy metal pollution. It is believed that increasing tillage intensity leads to more greenhouse gas emissions from soil [21,22] but less tillage erosion and crop yield [23]. Among the tillage measures, no-tillage has no effect on the stability of yield [24]. It builds soil fertility by increasing biological activity, reducing the need for fertilizers and minimizing their effects on the environment. It also reduces soil erosion, improves soil and water quality, increases crop yields, helps to sequester carbon and reduces labour [25,26]. Besides, it provides positive environmental benefit [27] and improves water use efficiency (WUE) of crops [28]. Shallow tillage (depth no more than 15–20 cm [29]) can reduce soil erosion, soil compaction, soil carbon loss, and soil evaporation [30]. It can also increase crop yield more than deep tillage [31]. Studies on mulching suggest that it prevents soil carbon loss and soil erosion [32]. Straw mulching increases soil organic matter content [33]. It is generally believed that the yield of mulch tillage is higher than that of conventional way [34]. However, there are also variations among different regions. In the Loess Plateau in China, plastic and straw mulching both improve the WUE of crops and increase yield [35], while in the North China Plain, mulching decreases crop yield compared with conventional tillage [36]. Therefore, the selection of covering materials should follow the principle of adapting to local conditions. Coupling of water and fertilizer achieves the effect of increasing production according to the matching ratio of water and fertilizer. The addition of different combinations of water and fertilizer can not only improve the yield, but can also significantly change the efficiency of water and fertilizers [37,38], reducing the accumulation of heavy metals in soil [39], and increasing soil water content and crop yield [40]. Poor water and fertilizer is not enough to enhance crop yield, but too much will cause unnecessary waste [41]. Chemical regulation is to develop water-retaining agents with water-absorption-and-retention capability as needed [42], which can improve soil porosity, increase soil organic matter, enhance crop yield and the WUE [43]. Crop allocation mainly adopts high-short and deep-and-shallow-root collocation to avoid competition for soil water and fertilizer, which is benefitial for crops to maximize the use of light, heat, water and fertilizer resources, and harvest more crop.

The above researches show that the study of agronomic measures has achieved a lot of fruitful results, and has made an important contribution to the increase of crop yield and the improvement of ecological and environmental benefits in various regions.

However, few studies have specifically investigated the suitability of agronomic water-saving technologies in karst areas. Based on the uniqueness of "karst drought", the "dual" spatial distribution of water resources and the discontinuity of soil distribution, this study clarified the suitability of agronomic water-saving measures such as tillage, mulching, water-fertilizer coupling, chemical regulation, crop allocation and deficit irrigation in karst areas, and found the existing problems. It proposed the effective way of strengthening the research and development of soil and water leakage monitoring and control technology, constructing the three-dimensional optimal allocation model of forest, grain and grass, and studying the "five-water" transformation theory from the watershed scale. The purpose is to realize the efficient utilization of water resources and the development of water-saving value-added eco-derived industries in the control of karst desertification.

## 2 Agronomic water-saving technologies

### 2.1 Tillage water-saving technology

There exists difference in the water-saving benefits of different tillage methods, such as ridge tillage, deep tillage and deep loosening, inter-tillage, less tillage and no tillage. Ridge tillage can thicken the soil ploughing layer, retain soil water and nutrients, enhance soil water storage capacity, promote water absorption by crop roots, and improve the WUE [44]. Wang et al. [45] conducted a study on cross-slope ridge tillage of crops in the slope cultivated land in Guizhou, China, and found that soil water content increased by 2.9 %–4.5 %, maize root distribution increased by 15.3 cm, and maize yield increased by 5.45 % compared with those in flat slope cultivated land. Deep tillage and deep loosening can break the plow pan, loosen the soil, increase the thickness of the plow layer [46], reduce the soil bulk density [47], increase rainfall infiltration and soil water storage, and improve the stability of soil aggregates, thereby reducing surface runoff [48] and improving crop yield and the WUE. Zhao et al. [49] conducted deep tillage and deep loosening research in the Huang-Huaihai area in China on the basis of straw returning to the field and found: Compared with conventional tillage, the soil bulk density in the 20–40 cm soil layer was reduced by 5.1 % and 6.6 %, the inter - plant evaporation was decreased by 22.7 % and 21.8 %, and the WUE was increased by 15.1 % and 15.9 %, respectively. Inter-tillage mainly cuts the connection between the surface layer and the underlying soil during the growth period of crops, prevents the underlying soil from providing water to the upper soil, reduces the soil-atmosphere interface water transport flux, inhibits the ineffective evaporation of soil water, and promotes rainfall infiltration and storage [50,51]. Zero or less tillage has a small disturbance to soil, which can reduce soil bulk density, increase organic matter content, promote high crop yield [52], and enhance soil water content [53].

In karst desertification areas, the soil distribution is discontinuous and the soil layer is shallow. In the past (about 20 years ago), people rarely left off their hometown for work in cities, and thus, there were sufficient labor force but less land. Ridge tillage was adopted by most people through gathering the soil to the roots of crops, which was conducive to the crops absorbing more water and fertilizer. Coupled with the application of organic manure, ridge tillage yielded more crops than less or no tillage. Because most of the crops in the same place received the same rainfall, the larger the yield was, the higher WUE achieved. Therefore, ridge tillage helped to improve the WUE of crops. At present, the labor force in the karst area is mostly migrant workers, and the local labor force is scarce, so that herbicides are widely used. The farming methods have changed compared to the past, and more people choose less or no tillage. As long as enough fertilizer is applied to crops, in good weather, the yield is even higher than before, and the WUE is also improved. To save labor force and improve the WUE, less or no tillage is suitable for the karst desertification area.

### 2.2 Mulching technology

**Fig. 1:**
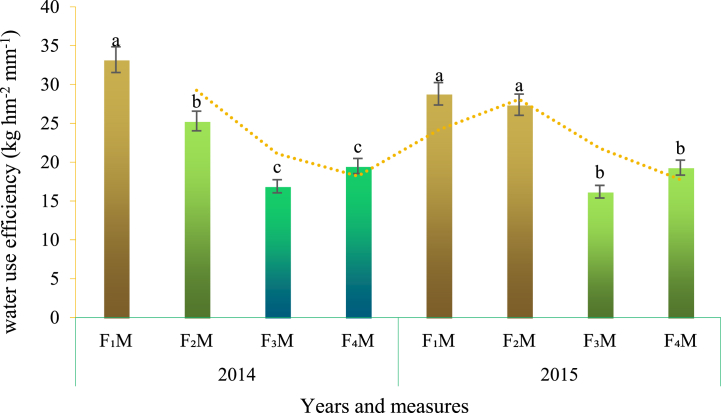
WUE with different mulching methods. F_1_M: Ridge-furrow planting with complete plastic film mulching; F_2_M: flat-planting with complete plastic film mulching; F_3_M: flat-planting with half plastic film mulching; F_4_M: Flat-planting without mulching (Control). Different letters indicate significant differences (P < 0.05).

Mulching is a traditional measure of agricultural water retention. Different covering materials are used to block the vertical evaporation of soil water, so that water can migrate horizontally, increase the water storage capacity of soil ploughing layer, and improve the water absorption conditions of crops. Mulching technology mainly includes mulching film, straw, sand and gravel. The traditional mulching cultivation technology appeared in China in the middle of the 6th century, while mulching film didn't appear until the middle of the 20th century and was first applied in developed countries such as Japan and the United States [54]. Sand and gravel mulching is a kind of water-saving farming method formed after long-term production practice in the arid area of northwest China. Compared with it, there are, currently, more studies on plastic film and straw mulching. Film has the effect of water retention and temperature holding [55]. With mulching film, the water vapor evaporated from the soil remains between the soil layer and the film, and after cooling at night, it liquefies into water droplets and then infiltrated into the soil layer, forming a small water cycle. Straw mulching is to cover the soil surface with crop branches and fallen leaves to change soil surface roughness, inhibit soil water evaporation and reduce surface runoff [56,57], which is conducive to crop growth and development, and improve the WUE [58]. The water-saving effect of mulching is affected by mulching type, mode, period, duration and amount, and is also related to crop type and test area [[59], [60], [61]]. Different covering methods brings different WUE. According to the experiments of different covering methods in Gansu Province, China, the highest WUE was F_1_M in 2014, and the lowest was F_3_M in 2015. In 2014, the WUE of F_1_M, F_2_M and F_3_M had significant discrepancy while the differences between F_3_M and F_4_M were not. In 2015, there were significant differences in the WUE under the measures of F_1_M and F_2_M from that under F_3_M and F_4_M (Fig. 1) [60].

**Fig. 2:**
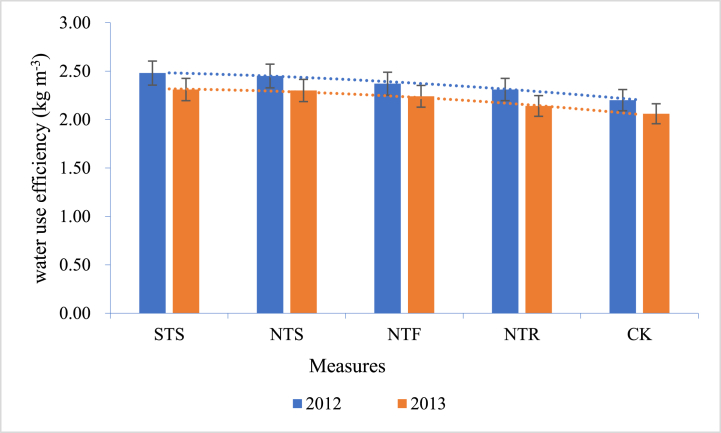
WUE with different measures.

The WUE of different straw mulching is also different. Wang et al. [62] conducted straw mulching experiments on maize fields in Harbin, China. They adopted the following models: surface tillage with straw mulching (STS), no-tillage with straw mulching on furrow (NTF), no-tillage with stubble mulching (NTS), and no-tillage with straw mulching on ridge and furrow (NTR). The test results showed that the WUE of all straw mulching was higher when compared with traditional tillage (Control group, CK) (Fig. 2). It is also confirmed in Wang et al. ’s monitoring experiments on different straw mulch in Liaoning, China, that compared with traditional tillage, the WUE of crops was improved to varying degrees [63].

**Fig. 3:**
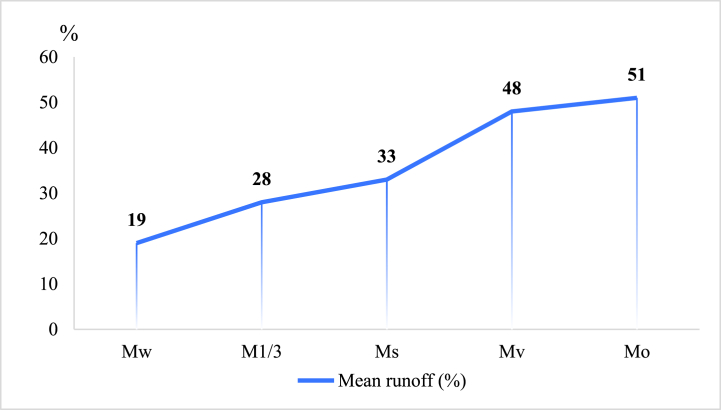
Effect of tillage and mulch application on runoff M_w_: straw mulch applied on the whole plot; M_1/3_: straw mulch applied on lower one-third of the plot; Ms: straw mulch applied in strips; M_v_: vertical mulching; Mo: no mulching (control).

Some scholars have also made comparative studies on plastic film and straw covering. The comparative study by Chen et al. [64] focused on the WUE of maize covered by plastic film and straw in Ningxia, China. It found that the WUE under plastic film of covering increased by 12.55 %–54.70 % compared with that of bare land. The water consumption of caragana microphylla (*Caragana Korshinskii Kom*) straw mulching was higher, but the WUE was relatively low. This possibly lies to its competition for water and fertilizer with crops during straw decay, smaller temperature difference between day and night in soil, imbalance of carbon and nitrogen ratio, and seed decay caused by straw obstruction [65]. An effective way to improve the WUE was to increase the mulching years [66]. Wang et al. [67] compared effects of plastic film and straw mulching on summer maize in Shaanxi province in China and found similar water retention effect by the two ways. The study by Pu et al. [68] in Shanxi Province in China showed that straw had a better water retention effect than plastic film. In the research by Wang et al. [69] in Gansu Province, China, the result showed that the WUE of spring wheat covered by straw was 17.17 %–43.01 % higher than that covered by film. In India, Bhatt et al. [70] studied the effects of straw and film mulching on soil erosion, and they found that straw covering reduced the runoff by 33 % compared with non-mulching, and the water retention effect that of plastic film mulching (Fig. 3). It has been suggested in most studies that the water retention efficiency of straw mulching is better when compared with film mulching, especially in dry-farming regions under drought conditions [65].

In the karst desertification area, mulching film is mostly chosen for planting cash crops, such as *flue-cured tobacco* mulching film and dragon fruit (*Hylocereus undulatus Britt*) mulching film, mainly in order to retain water, increase soil temperature, and improve yield and economic benefits. Mulching film is simple and convenient, but there are still some problems. First, the film is difficult to degrade, easy to cause soil environment pollution; second, the film is usually applied to ridge planting. When it rains, the rain water flows along the plastic film to the furrows, causing the lack of water in the soil of the plant roots on the ridges. In order to ensure the crop roots receiving rain water, people have to tear the film above the crop roots, which fails to bring the benefit of film coverage, but leads to waste of mulching film. In recent years, in order to save costs, people have changed the traditional planting methods, on the basis of no and less tillage, to carry out full cover with the maize straw and wheat straw. Maize and winter wheat are the two main food crops in the karst desertification area. Yet in recent years, winter wheat has been seldom planted, so the straw is mainly from maize. No-tillage with full coverage of straw means: After harvesting maize in autumn, the maize stalks are completely covered in the land for decomposition and decay, and the next spring, seeds are planted in the covered land through hole sowing. Karst area belongs to the engineering dry area, and like other dry places, straw mulching also has the best water retention benefit. When applying this measure, straw should be crushed into appropriate size according to the actual situation of local temperature and precipitation, so as to ensure that the straw can be fully decomposed during spring sowing. This measure will enhance soil organic fertilizer, build soil fertility, improve crop yield and the WUE.

### 2.3 Water-saving technology of water and fertilizer coupling

In agricultural production, water and nutrients are not isolated, but interactive and mutual influential. With limited water resources, the implement of water-fertilizer coupling research is an effective way to realize soil water increasing by fertilizer and fertilizer promotion with water. This measure improves water resource utilization efficiency, and promote sustainable agricultural development [71,72]. At present, the hot issues facing water-saving agriculture are how to regulate the temporal and spatial distribution of water and nutrients, explore the optimal ratio of water and fertilizer, and more efficiently utilize water and fertilizer [73]. Scholars have carried out a large number of studies on the coupling models and effects of water and fertilizer in different soil environments and crops in different regions. The effect of yield increase under three factors of nitrogen, potassium and water on crops is: Water is better than nitrogen, which is superior to potassium, and water is an important limiting factor for the coordination of the two factors. When there was sufficient water, appropriately increasing the amount of nitrogen and potassium can increase yield and achieve the purpose of improving the WUE [74]. Nitrogen and phosphorus fertilizer application under drought stress was beneficial to improve crop WUE [75]. The WUE of cotton in Xinjiang in China was the highest when the water and fertilizer ratio of cotton was 362.3–462.5 mm and N–P_2_O_5_–K_2_O was 212.5–85–42.5 to 367.5–147–73.5 kg ha^−1^ [76]. In the coupled treatment of winter wheat in Guangdong Province, China, under the same water consumption, the WUE was increased by 34.03 %, 13.35 %, and 20.86 % in the UNS2 (urea: slow-release N fertilizer = 1:3) treatment compared to U (100 % urea), UNS1 (urea: slow-release N fertilizer = 1:1), and SRF (100 % slow-release N fertilizer), respectively [77].

In karst desertification areas, the common practice is to apply base fertilizer at the time of sowing. Previously, the base fertilizer was mostly organic fertilizer, and now almost all are chemical fertilizer (compound fertilizer). After the base fertilizer is applied, once the rain falls, nitrogen, phosphorus, potassium and other elements in fertilizer dissolve into ionic water fertilizer when encountering water, which can be absorbed by seeds and seedlings. Nevertheless, the soil has no enough nutrients to supply the growth of crops in the crop growing period, and it is also necessary to perform top application. It is very important to control the topdressing time. Once the fertilizer (mostly compound fertilizer) is applied to the roots of the crops but without rain for a few days, the fertilizer will evaporate and become inoperative. Therefore, people need to apply topdressing within a few hours before rainfall, and the fertilizer will encounter rain soon, resulting in the coupling effect of water and fertilizer. This will provide nutrients for crops, promote the utilization rate of water and fertilizer, and improve crop yield and the WUE.

### 2.4 Chemical regulation of water-saving technology

Chemical regulation technology uses chemical agents to regulate water to achieve water saving. Polyacrylamide (PAM) can be used to regulate the surface soil structure. It can also be realized through the water and fertilizer retention and slow release performance of super absorbent polymer (SAP) and its regulating effect on the root layer soil structure. Besides, the anti-transpiration agent fulvic acid (FA) can be used to regulate the growth of crops [78]. The study and use of water-retaining agents in China began in the 1980s, and are currently mainly applied in agriculture and forestry fields [79]. The water-retaining agent is composed of water-absorbing resin with a three-dimensional network structure of molecules and a large number of hydrophilic groups. It has super ability of water absorption and moisture retention. After absorbing water, it expands into hydrogels, and then slowly releases water to crops for use. It is non-toxic and non-irritating [80], being able to increase the number of soil aggregate with mechanical stability [81], promote soil water storage [82], and inhibit soil water evaporation. The components of plant anti-transpiration agents contain low molecular weight humic acid, which is easy to be absorbed by crops, and can promote stomatal contraction of plants, reduce leaf water potential, maintain water in plants, inhibit leaf surface water evaporation, and reduce partial ineffective transpiration of crops, thereby reducing water consumption of crops and enhancing drought resistance.

The WUE of crops is affected by the amount and depth of different water retaining agents. Zhang et al. [83] applied water retention doses of 30, 37.5, 45 and 52.5 kg ha^−1^ to tomatoes in Inner Mongolia, China, and compared them with the control group. It was found that the WUE increased by 19.1 %, 50.4 %, 81.5 % and 69.2 %, respectively. Zhang et al. [84] conducted an experiment in Jilin Province, China, and the results showed that when the application depth of water retaining agent was 10, 20, 30 and 40 cm, the optimal application depth of different water retaining agents was different. The optimal application depth of MP3005KB (particle size: 0.3–0.8 mm) was 30–40 cm, but 20–40 cm for US (particle size: 0.01–0.02 mm) and XM (particle size: 0.8–1.6 mm). The WUE was the highest when the application depth of water retaining agent reached 40 cm (Table 1).

**Table 1:** Effect of application depth of water retaining agent on the WUE of maize.

	Depth of application (cm)	MP3005KB	US	XM	CK
WUE (kg m^−3^)	10	3.12	2.98	3.28	3.5
	20	3.25	4.02	3.84	/
	30	3.95	4.21	3.99	/
	40	4.17	4.68	4.39	/

In karst desertification areas, obstacles for water retaining agents still exist. First, the soil layer is shallow, less than 10 cm in many places, failing to provide the optimal application depth of soil water retention agent. In addition, people in karst areas live dispersedly, and the land they own is also very scattered (most are slope farmland), which presents difficulty to bring water retaining agent to their own land. Third, the preparation of water retaining agent is too complicated. As a result, the application of water retaining agent only stays on the stage of laboratory pot test or field test.

### 2.5 Water-saving technology of crop allocation

**Fig. 4:**
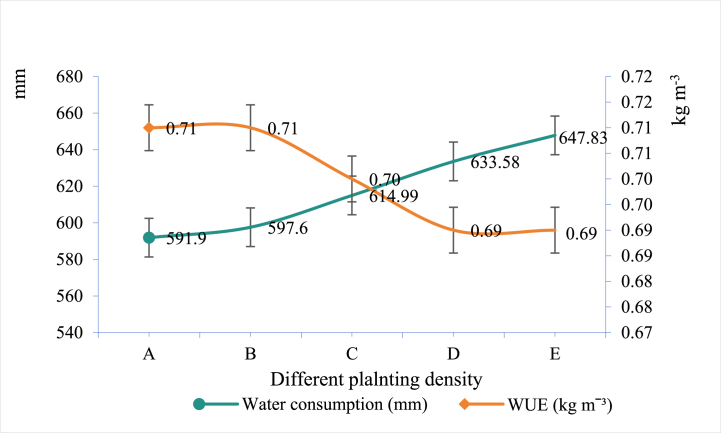
Water consumption and the WUE of sunflower during the whole growth period Note: A/B/C/D/E in the figure represents different planting density of sunflowers in the wheat and sunflower intercropping systems as 4/5/6/7 plants per square meter, respectively.

Water saving by crop allocation is to choose water - saving and yield - increasing crops according to local conditions and rationally arrange the planting layout of crops. Selecting water - saving and value - added species refers to choice of drought - tolerant and high - yield crops suitable for the climate and soil conditions of the planting area. Adjusting planting layout is to adjust the planting arrangement of crops in temporal and spatial in condition of sunlight, heat, water, fertilizer and so on. To develop water - saving agriculture, it is necessary to rationally arrange the planting layout according to the amount of rainfall, appropriately reduce the planting area of crops with high water consumption, prevent the unification of planting species, and mix high - short, deep - rooted and shallow - rooted crops [85]. By adopting inter - cropping, rotation, and mixed cropping, we can make full use of sunlight, heat, water and fertilizer resources, reduce the survival competition pressure of crops, maximize the utilization rate of land resources and water resources, and improve the production efficiency of farmland. The tall and short allocation makes the most of sunlight and heat resources, while the combination of deep and shallow roots can reach maximum absorption of water and nutrient in all layers of soil and thereby weaken the competition of water and fertilizer. Wei et al. [86] studied the effects of different planting densities on the WUE under wheat interplanting sunflowers in Inner Mongolia, China, and found that increasing the planting density of sunflowers could improve the WUE, and the water saving effect was the best with the density of 5 plants per square meter. The water consumption of interplanting sunflowers increased significantly with planting density of sunflowers, while the WUE presents a unimodal curve (Fig. 4). Ding et al. [87] intercropped wheat and maize in Gansu province, China, and it was found that water was able to be saved by 9.85 % when the planting density was appropriately increased. Therefore, properly increasing the planting density of intercropping mode helps to bring water-saving and value-added benefits.

Water - saving species in karst areas mainly include drought - resistant ones and crops with low water consumption. The reason for such selection is that there exists not only ‘karst drought’, but also spring and summer drought. Drought has long hindered the growth of crops and plagued the economic development, and thus drought resistance is the guarantee of normal crop growth. Water consumption of crops mainly refers to evapotranspiration, and less water consumption is reflected in the reduction of crop transpiration rate and soil evaporation. By shading each other, agroforestry reduces crop transpiration rate and soil evaporation, hence meeting the requirements of water-saving through crop allocation in karst areas. The perennial woody plants in agroforestry can be cultivated by dwarf and dense planting to minimize the luxury transpiration of crops to lower their water consumption, while the planting density of crops can be appropriately increased to maximize the WUE of agroforestry.

### 2.6 Deficit irrigation

Deficit irrigation is an agronomic measure in which the irrigation water in the drought-sensitive growth stage of crops is lower than the maximum crop yield water requirement [88]. The irrigation amount is lower than the normal evapotranspiration of crops [[89], [90], [91], [92]]. This measure aims to produce no or less effect on the yield on the premise of diminished the water consumption [[93], [94], [95]], and then to improve the irrigation WUE and crop WUE [[96], [97], [98], [99]]. Currently, the irrigated agriculture has moved from the traditional single pursuit of yield to the stage of simultaneous enhancement of yield and WUE [100]. Accordingly, people often combine the deficit irrigation water-saving method with fertilizer, hydrogel and mulch tillage to form a composite agronomic water-saving and yield-increasing measure, expecting to obtain more net income and better crop quality. On the Loess Plateau of China, the yield of summer corn was reported to increase by 1.8–2.3 % under the composite measure of deficit irrigation +24 % organic fertilizer +76 % inorganic fertilizer when compared with only inorganic fertilizer, and the WUE was well improved [101]. In Nanjiang region of Xinjiang, China, the experiment of applying deficit irrigation + film coating to Cyperus esculentus (*Cyperus esculentus* L. *Var. Sativus Baeck*) revealed that the WUE, dry matter amount and quality (crude fat, crude protein, soluble starch and soluble sugar) of Cyperus esculentus under the measure of mild deficit irrigation + mulching were higher than those without mulching [102]. In the greenhouses of Luancheng District Agricultural Planting Base in Shijiazhuang City, China, experiments were conducted and the result showed that deficit irrigation with increased silicon fertilizer was beneficial to tomato growth and quality improvement [103]. It was also reported in Kenya that the composite measure of 50 % deficit irrigation + cover tillage, in watermelon (*Citrullus lanatus*) planting, saved 262.75 mm of water and increased the yield by 27.90 % when compared with that of non-cover tillage +100 % full irrigation [104]. In addition, there are studies saying that diverse soil textures affect the benefit of deficit irrigation. In Egypt, lettuce was planted in three types of soil with different textural classes (clay, loamy sand, and sandy clay soil), and treated with measures of hydrogel in various concentrations (0, 0.1, 0.2 and 0.3 % w/w) + deficit irrigation. It was found that the lettuce reached the maximum fresh weight in clay and the minimum in loamy sand, with the medium in sandy-caly soil, indicating that the response of crop yield and WUE to deficit irrigation varies in different soil texture [105].

Although karst areas feature the rain-fed agriculture, seasonal drought (spring drought or summer drought, or spring drought followed by summer drought) seriously hinders crop growth. Yet the scattered land in karst mountainous areas prohibits adequate irrigation. In the hot-dry valley areas where spring and summer droughts often occur, such as Zhenfeng-Guanling-Huajiang Medium-Intensity Karst Desertification Control Demonstration Zone in Guizhou, China, many facilities such as road surface rainwater collection, roof rainwater collection and rainwater collection ponds have been built to realize comprehensive control of karst desertification and thereby develop eco-derived industries. Nevertheless, many collection tanks built before were abandoned, for the reasons that the land was usually too far away from the water intakes or the rainwater collection tanks failed to collect water well, along with the similar amount of rainfall and evaporation [5]. Consequently, the alternative and effective way is to build simple ecological wading ponds in the farmland to collect rainwater. Yet this way still face its challenges: less collected rainwater, over evaporation. In the real practice, therefore, people have to transform the ecological wading ponds to reduce the evaporation without reducing the amount of rainwater that can be collected, such as placing a dome cover on the pond. When crops are under drought stress, deficit irrigation is carried out with the limited water from the ecological ponds.

## 3 Problems and countermeasures to be concerned in agronomic water-saving technology in karst areas

### 3.1 In view of the optimal model of water-fertilizer coupling under different niches and planting patterns in karst areas, a large number of water-fertilizer ratio tests should be conducted to establish a regional long-term monitoring, explore the mechanism of water-fertilizer coupling, and build a regional water-fertilizer coupling model

The variation of region, climate, soil environment and planting methods makes it difficult for the optimal ratio model of water and fertilizer coupling in a certain place to be demonstrated and promoted in other places. The experiment by Zhang et al. [106] on the ratio of water and fertilizer to maize showed the optimal water and fertilizer mode was as follows: water of 700 m^3^ ha^−1^, nitrogen fertilizer of 270 kg ha^−1^, phosphate fertilizer of 60.26 kg ha^−1^ and potassium fertilizer of 60.02 kg ha^−1^. Ma et al. [107] found the optimal water and fertilizer mode of maize as follows: water was 848.24 m^3^ ha^−1^ and fertilizer application amount was 192.66 kg ha^−1^. In the above two studies, the optimal ratio of water and fertilizer coupling is sharply different though they both aimed at maize planting. This suggests the difficulty to popularize an optimal mode in another region. Even for the same crops in the same region, influenced by changes in meteorological factors such as rainfall and temperature, there is discrepancy in the demand for water and fertilizer for crops in different years. Wu et al. [108] found that under the same conditions and in terms of yield, the optimal water and fertilizer coupling mode in 2012 was irrigation amount of 425 mm, N fertilizer of 310 kg ha^−1^ and P_2_O_5_ 124 kg ha^−1^. The optimal model for K_2_O 62 kg ha^−1^ in 2013 was 392 mm irrigation volume, N fertilizer of 281 kg ha^−1^, phosphate fertilizer (P_2_O_5_) of 112 kg ha^−1^ and potassium fertilizer (K_2_O) of 57 kg ha^−1^, all relatively lower than those in 2012. In karst areas, niche habitats are complex and diverse, with heavy soil heterogeneity. Most of them are slope cultivated land. The elevation difference between the hilltop and the foot of the slope will cause changes in rainfall, temperature and other meteorological factors, which will further affect the water-fertilizer ratio of crops. Nevertheless, it is impossible for us to develop a water-fertilizer coupling model based on every little variation. We can only take a certain region as a unit (such as a basin or county with similar meteorological factors), establish a long-term monitoring through a large number of water-fertilizer coupling tests, explore the water-fertilizer coupling mechanism of major food crops and cash crops, and construct the optimal model of water and fertilizer coupling to obtain the highest WUE and maximum crop yield.

### 3.2 In view of the single application of agronomic water-saving measures in karst areas, it is advisable to adopt composite measures and carry out integrated demonstration and promotion of technologies

Each agronomic measure has its own advantages and disadvantages. Straw mulching can improve the WUE and yield [109], increase the contents of potassium, calcium, magnesium, phosphorus and boron in the soil [110], enhance soil fertility, reduce soil evaporation [111], and alleviate soil erosion [112]. Nonetheless, straw mulching also manifests some shortcomings. When we choose some relatively large straw, it cannot be decomposed by microorganisms in a short time and fails to quickly supplement the organic matter to the soil to enhance its fertility, thereby resulting in the soil nutrients unable to be normally supplied to the growth of crops and even soil pollution by the straw undecomposed by microorganisms in a long time. Plastic mulching is conducive to increase soil temperature, maintain soil moisture, improve soil quality [113], control weeds [114], promote crop growth, increase crop yield, and improve crop quality [115]. However, if the mulching film is not properly managed, a large amount of plastic waste enters the soil environment [116]. It is suggested here that whenever we lay out agronomic measures, we have to make the best use of the advantages and avoid the disadvantages. In karst areas, agronomic water-saving measures appear to be single. In most cases, mulching film is only applied to cover the critical cash crops in small areas, rarely using straw mulching, and less using both measures together. In the real practice in these areas, we can mash the residues after harvest into straw on the spot, and apply the principle of composting to pile them into piles for fermentation. In the planting season, the fermented straw will be used to perform mulching tillage, and then covered with mulching film. The straw returned to the field can supplement a large amount of soil organic matter and thereby improve soil fertility, while plastic mulching, on the other hand, can increase soil temperature and soil moisture content, and finally maximize the economic benefit. This composite agronomic water-saving technology requires more labor force, thus generally uneasy to be accepted by farmers. In view of this, a certain scale of land should be selected for demonstration and promotion, so that farmers can see that combined measures will obtain more economic benefits, and thereby make composite agronomic water-saving technology an important part of the development of water-saving agriculture.

## 4 Enlightenment of agronomic water-saving technology in the karst desertification control

Water resources provide the foundation for sustainable development of karst ecosystem. The major problems about water resources in karst desertification areas are engineering water shortage and low utilization efficiency of water resources. Solving the problem of water resources helps to promote the efficient development of local agriculture and forestry industries, increase vegetation coverage, form the ecological environment virtuous cycle. It can also bring social and economic benefits, and accelerate the comprehensive curbing process of karst desertification. The karst desertification area mainly has rainfed agriculture, presenting great potential to utilize the regional water resources through taking effective water-saving measures to enhance the utilization rate of rainfall. Agronomic water-saving measures have low cost, easy to be accepted by farmers and to be implemented. Nonetheless, to comprehensively control karst desertification from the perspectives of the ecological, social and economic benefits, it is necessary to fully consider the natural environment in the region, adapt to local conditions, and develop agronomic water-saving technologies suitable for regional environment and microclimate.

### 4.1 To construct agroforestry models with high-medium-low optimal allocation of forest-grain-grass according to the requirements of karst desertification control and development of ecological derived industries, as well as the characteristics of cultivated land in karst desertification areas

The control of karst desertification is not only to grow plants (such as planting shrubs and vines, which grow rapidly and have strong vitality, and can quickly cover the exposed stones), so that the distribution area of bare stones is reduced, but also to consider the people in these areas to get rid of poverty and get rich. Karst desertification is so serious an ecological environment problem (the earth's ‘cancer’, hard to control) that water-saving ecological derivative industry is necessary to be developed. Limited by such problems as bare rocks, less soil but more stones, discontinuous soil distribution and shallow soil layer, these areas require better use of limited land and hence agroforestry is a better choice in the development. Taking the perennial woody plants as basis, agroforestry has understory planting and breeding developed on the agricultural land, and thereby becoming an ecological industry that maximizes social, economic and ecological benefits on limited land. The forest-grain-grass model makes full use of the high-medium-low three-layer space of the land in the karst area. The upper ‘forest’ is generally the warp fruit forest (pear, Sichuan pepper, dragon fruit, walnut, roxburgh rose, etc.), and the major concern is the economic benefits of the ‘forest’. The medium layer is grain that mainly meets people's food needs, including soybean, maize, wheat, potatoes, peanuts and so on. The lower layer is grass (ryegrass, duck grass, etc) that helps to develop mountain animal husbandry, which is an important source of local agricultural economy since the karst desertification area is mountainous. The cultivation of agroforestry requires scientific management. When planting, it is necessary to consider whether the configuration of agroforestry is reasonable. In the growth process, the forest needs to be properly pruned, so as to reduce the transpiration, and increase the possibility of medium and lower crops receiving more rainfall and sunlight. If the grass in the lower layer is not properly managed, it will compete more with the trees and corn for water and nutrient. Therefore, when the grass grows to more than 20 cm in height, it should be cut to grow again, which increases the yield of grass and weakens its competition with forest and grain for soil moisture and nutrient. In addition, agroforestry can also form forest-bee system, forest-grazing system, forest-poultry system, thereby reducing water consumption on land, increasing economic benefits, and achieving the water-saving and value-added purpose.

### 4.2 According to the requirements of karst desertification control for soil and water loss and the effectiveness of underground soil leakage loss monitoring, the research and development of soil leakage loss monitoring and control technology should be strengthened

Karst desertification is mostly caused by soil erosion resulted from rock exposure. The key to control it is to reduce soil erosion. In other words, inhibiting soil and water loss is to make full use of them and prevent them from being lost to other places, thereby achieving efficient use of water resources and effect of water saving. In karst area, the special surface-underground dual geological structure causes soil and water loss from not only surface but also underground. Loss from the surface is easy to monitor while underground leakage is concealed and difficult to monitor. At present, the monitoring methods for underground soil leakage loss in all studies include simulation experiments, underground river monitoring method, scratching and erosion pins, underground water turbidity measuring, cave drip tracer method, and ^137^Cs tracer method [117]. Although with so many monitoring methods, it is a great challenge to preform accurate field monitoring. For many years, researchers have tried everything they can to solve this scientific and technological problem, yet the effectiveness of soil leakage loss monitoring has so far been limited to confirm the phenomenon of soil leakage loss existing, and it is still challenging to quantify the amount of loss.

Soil leakage loss control is based on quantitative monitoring technology. However, due to the lag of such technology, there are few studies on this subject. Some scholars proposed to develop ecological industry by planting dragon fruit + grass on slope to prevent and control soil leakage loss for the reason that dragon fruit has many long horizontal roots which can block slope cracks [118]. Some tried to preform pot incubation test in dolomite karst areas and the results demonstrated that adding water retaining agent with activated carbon can reduce soil leakage loss [119]. These studies, however, only stay at the phase of simulation and theory, not having been applied in the field combined with monitoring techniques. Therefore, the future research focus is to break through the research and development of soil leakage loss monitoring and control technology in the field, extend the simulation tests to the field, develop new technologies in an all-round way, combine bio-agronomic measures to prevent and control soil leakage loss, and develop water-saving and ecological industries.

### 4.3 According to the demand for the efficient use of water resources in the karst desertification control, the research on ‘five waters’ transformation at the basin scale should be strengthened

Karst area has mostly rain-fed agriculture. It requires the selection of drought-resistant species and a good grasp of the law of farmland hydrological cycle, thereby achieving highly efficient use of water resources in each process of farmland hydrological cycle to improve the WUE of crops. ‘Five waters’ consists of precipitation - surface water - groundwater - plant water - soil water. Precipitation is the total water resources, entering the hydrological cycle through surface, underground, soil and plant. The efficient use of precipitation is to collect rain on slope, road surface and roof in non-farmland areas. In farmland, it is reached by dwarf dense planting, pruning technology and tillage to reduce the amount of vegetation interception and increase soil infiltration, so that crops can absorb more soil water. The efficient use of surface water is to use different forms of water trapped on surface for the growth of farmland crops. The efficient use of soil water is mainly reflected in increasing soil infiltration and reducing soil evaporation. The efficient use of plant water refers to reducing vegetation interception and crop luxury transpiration under the premise of increasing crop yield.

No matter from the scale of farmland or basin, hydrological cycle should be studied from the perspective of system, which is exactly the object of five-water conversion study. At karst basin scale, affected by such factors as ground surface fragmentation, uneven plant density, a wide variety of species, and the great discrepancy in rates of soil evapotranspiration under different types of vegetation cover, it has big challenges to extend the study of five-water conversion from the scale of farmland to that of basin. Future studies should strengthen the experimental monitoring of five-water transformation at farmland scale, building a farmland hydrological cycle model. With the help of GIS technology, it is suggested developing a basin-scale GIS five-water transformation module, so as to achieve five-water transformation research at the basin scale, and thereby control the process of hydrological cycle from a macro scale, mastering the laws of hydrological cycle and crop water consumption to improve the efficiency of water resources utilization.

## 5 Conclusions and future directions

This study, through literature review, discussed the water-saving benefits of agronomic water-saving technologies, including tillage, mulching, water-fertilizer coupling, chemical regulation, crop allocation and deficit irrigation. It analyzed the suitability of agronomic water-saving measures in karst areas, presented the existing problems in agronomic water saving here, and revealed the significance of improving the utilization of soil and water resources for the control of karst desertification. The findings are as follows.

(1) Combining with the objective reality of labor force, land productivity and economic benefits in karst desertification area, and from the perspective of saving labor cost and ensuring efficient use of water resources, it is appropriate to adopt the farming method of less tillage and no tillage in karst area.
(2) On the basis of no or less tillage, the selection of corn straw mulching can increase soil fertility and improve the WUE of crops.
(3) Karst areas are dominated with rain-fed agriculture. When topdressing crops during the growing period, the coupling of water and fertilizer is mainly decided by the precise estimation of rainfall time by farmers, who need to put fertilizer on the roots of crops several hours before rainfall, and ensure that it will not be washed away by heavy rain and lost to other places.
(4) The complicated configuration process and scattered land make it difficult to apply and promote water retaining agents in karst areas.
5) In order to maximize social and economic benefits with limited site space, agroforestry is suitable for water-saving crops in karst areas, and the forest needs to be dwarfed and densely planted to properly increase the planting density of crops, which can improve the WUE of crops.
(6) With heavy soil heterogeneity and significant differences in meteorological factors in karst areas, long term monitoring can only be conducted based on a large number of water and fertilizer ratio experiments to explore the coupling mechanism of water and fertilizer between major food crops and cash crops in the region. The optimal water and fertilizer ratio model for crop WUE and yield is waiting to be constructed. Combined agronomic water-saving technology (mainly straw mulching and mulching film) can be used for demonstration and promotion to improve the utilization efficiency of water and fertilizer under mulching.
(7) In the control of karst desertification, it is necessary to strengthen the research and development of soil leakage loss monitoring and control technology, carry out comprehensive soil and water loss regulation, strengthen the research of five waters transformation at the basin scale, grasp the law of crop water consumption in different periods of crop growth, and develop water-saving and value-added ecological derivative industries of agroforestry with high-medium-low optimal allocation of forest, grain and grass.

The analysis of agronomic water saving in this study is based on experimental cases through literature review. The studied literature covers only one or several years. However, the development and efficiency of agronomic water-saving technologies will change with the variation of transportation means, land use mode and climate. To be more reliable, future researches on agronomic water-saving measures are expected to be conducted on a certain region and extended to 20, 30, 50 years or longer time.

## Data availability statement

The data that support the findings of this study are available from the corresponding author, [Qinglin Wu], upon reasonable request.

## CRediT authorship contribution statement

**Qinglin Wu:** Writing – original draft, Software, Resources, Project administration, Methodology, Funding acquisition, Formal analysis, Data curation, Conceptualization. **Lan Wang:** Writing – review & editing, Validation, Project administration, Funding acquisition, Conceptualization.

## Declaration of competing interest

The authors declare that they have no known competing financial interests or personal relationships that could have appeared to influence the work reported in this paper.
